# SOD2 genetics regulating mitochondrial management of oxidative stress is tied to chemical sensitivity in Gulf war veterans

**DOI:** 10.1038/s41598-025-09916-w

**Published:** 2025-07-08

**Authors:** Beatrice Alexandra Golomb, Leeann Bui, Brinton Keith Berg

**Affiliations:** 1 Department of Medicine, UC San Diego School of Medicine, 9500 Gilman Drive #0995, La Jolla, 92093-0995 CA USA; 2 Present Address: OBGYN Department , Santa Clara Valley Medical Center , San Jose, CA USA

**Keywords:** Diseases, Medical research, Risk factors

## Abstract

Multiple chemical sensitivity (**MCS**), though viewed by many as psychogenic, was presumptively tied to genetic variation in superoxide dismutase 2 (**SOD2**), involved in conversion of mitochondrial superoxide to hydrogen peroxide, in Japanese paper pulp workers. Gulf War veterans (**GWV**) have increased MCS compared to nondeployed persons. In data from GWV and nonveterans, we assessed whether altered oxidative stress management via SOD2 polymorphism was tied to self-rated chemical sensitivity. Sixty white predominantly male GWV and age-similar nonveterans completed self-ratings of chemical sensitivity, and underwent both nuclear DNA analysis for SOD2 variants and mitochondrial haplogroup assessment. SOD2 Ala16 (vs. Val16) significantly predicted self-rated chemical sensitivity in GWV and in the total sample (ordinal logistic regression with robust SEs): OR(SE)[95% CI] = 3.56(1.83)[1.30, 9.74], *p* = 0.013 (total sample); OR[95% CI](SE) = 6.36(4.85)[1.42, 28.4], *p* = 0.015 (Gulf-deployed). Significance was sustained with adjustment for mitochondrial haplogroup U (not itself significant) and when reappraised with nonparametric trend tests (Kendall’s Tau, Spearman Ranked Correlation Coefficient). The findings extend evidence of SOD2 polymorphism ramifications for chemical sensitivity to a new chemically exposed sample, implicating mitochondrial oxidative stress management as a (though not necessarily the exclusive) key factor in chemical sensitivity. Findings comport with burgeoning evidence inculpating mitochondrial impairment and oxidative stress in many drug/chemical/environmental factors’ toxicity irrespective of the agent’s nominal mechanism of action. This supports chemical sensitivity as a physiological, not a psychogenic condition.

**Supplementary Information:**

The online version contains supplementary material available at 10.1038/s41598-025-09916-w.

## Introduction

Multiple chemical sensitivity (**MCS**), also termed “toxicant-induced loss of tolerance,”^1^ refers to a condition in which individuals – typically following a significant toxicant exposure – report becoming symptomatic following low-level exposure to any of many substances often of widely disparate chemical class and nominal mechanism of action. Many presume this to be implausible physiologically, so ascribe MCS to psychogenic causes. This is reflected in the coding of multiple chemical sensitivity among the somatoform disorders under diagnostic code F45 in the International Classification of Diseases (ICD-10)^2^. However, mitochondrial impairment and oxidative stress could provide an alternative explanation. Chemicals with mitochondrial and oxidative toxicity can impair mitochondrial function and elevate free radical production^3–5^. Perhaps particularly if the damage extends to mitochondrial DNA^6^, which has limited repair capacity, this may result in sustained dysfunction, lowering the threshold for symptoms in response to future chemical exposures that themselves impair energy or promote oxidative stress – as many chemicals can do^7^.

Meanwhile, superoxide dismutase 2 (**SOD2**), also called manganese superoxide dismutase, converts mitochondrial superoxide to hydrogen peroxide, and has been termed the principal mitochondrial antioxidant^8,9^. There are two common variants of SOD2 based on a C vs. T substitution at position 47 in the DNA sequence^10^, leading to an alanine vs. valine substitution at amino acid position 16 in the resulting SOD2 enzyme. In humans, genetic polymorphisms of SOD2 have been reported to modulate oxidative stress status^11^. Different populations have different ratios of Ala16 to Val16: Europeans are balanced at ~ 50% each, while East Asians show predominantly Val16 (86%), and Latin Americans of primarily European and Native American descent (not Afro-Caribbean) show primarily Ala16 (61%)^12^, underscoring importance of genetic homogeneity in evaluating gene-outcome associations for SOD2. Distinct selection pressures, including other genetic or lifestyle factors, could influence both selection and implications of gene variants. Both the terminology SOD2 A16V and SOD2 V16A are in use^13–21^: Each imply that the first designated amino acid is the “reference” allele with the second named amino acid the replacement being studied. Each variant can produce worse mitochondrial oxidative stress status in different settings^22^. Oxidative injury may be promoted with deficient or with excessive SOD2 activity (the latter if clearing of the hydrogen peroxide products – by catalase, glutathione peroxidase or peroxiredoxins – is sluggish and/or elevated iron promotes hydroxyl radical formation from it, for instance), in each case expected to be more problematic in settings of greater mitochondrially toxic and/or oxidative stress promoting exposures. The Ala16 variant has been tied to more SOD2 in the mitochondrion, but is also tied to greater oxidative stress in some settings. A further factor is that iron can substitute for manganese in SOD2 to convert the enzyme to a prooxidant form^22^. A Japanese study of 324 individuals who worked at a paper pulp producing company, of whom 116 had chemical sensitivity (36%), reported that having the Ala16 allele was significantly related to chemical sensitivity – particularly more severe MCS^23^. The Ala16 allele is tied to higher levels of SOD2 within the mitochondrion. But, as above, whether this is favorable or adverse for mitochondrial oxidative stress varies^22^.

Whether the SOD2 Ala16 allele is tied to chemical sensitivity, and particularly to more severe chemical sensitivity as in the Japanese study, in a different high-chemical exposure high-chemical sensitivity sample, and/or in individuals of different ethnicity has yet to be examined. Gulf War deployment – in the 1990–1991 Operations Desert Shield and Desert Storm – provides a promising setting to evaluate an SOD2 genetic variant association to chemical sensitivity. The conflict involved an unprecedented array of new, unique, and excessive exposures, most if not all with potential for mitochondrial and/or oxidative toxicity. Exposures included, but were not limited to, acetylcholinesterase inhibiting carbamates and organophosphates^24–26^. This occurred via carbamate and organophosphate pesticide exposure^25,27^, exposure to the carbamate nerve agent pretreatment adjunct pyridostigmine bromide (**PB**)^24^, and exposure to organophosphate nerve agents (sarin and cyclosarin)^28,29^. Both organophosphates and carbamates, for instance, have mitochondrial and oxidative stress toxicity and these agents meet Hill’s presumptive criteria for causality as a contributor to Gulf War illness (**GWI**)^26^. Additional supportive evidence of a relationship to these agents comes from gene-environment interaction findings^30–32^. Consistent with these agents being implicated, GWI presence and severity have been shown to relate to level of mitochondrial impairment^33–35^. However, a causal relation for these does not mean these are the only causal factors – both for the agents and the proposed mechanism – and there is interest in other mechanisms such as neuroinflammation^36^ and autoantibody induction^37^, though these themselves can be promoted by oxidative stress and mitochondrial impairment^34,38–41^. Other notable exposures include oil well fires; permethrin impregnated uniforms; anthrax vaccine; botulinum toxoid vaccine (unique to that conflict); an unprecedented number of multiple vaccines; first use of depleted uranium employed to gird tanks and munitions, bearing heavy metal and low-level radioactivity toxicity; use of ciprofloxacin, a fluoroquinolone antibiotic, in some instances for reported biowarfare prophylaxis; jet fuel; burn pits; chemical agent resistant compound (**CARC**) paint; as well as the finest sand in the world – down to < 0.5 microns diameter, capable of penetrating the alveoli and carrying chemicals and pathogens into the lung; and extremes of heat and cold^42–44^. In contrast to later conflicts, the ground war only lasted four days, many never saw combat, and combat stress does not show an independent relation to GWI^45^. Virtually all of these exposures can contribute to oxidative and/or mitochondrial toxicity^46–85^. For several of these agents where direct investigation of oxidative stress and mitochondrial toxicity has not encompassed the agent per se, citations provided are for a component of the agent, such as aluminum use as an adjuvant in the anthrax and some other vaccines, formaldehyde in the botulinum toxoid vaccine, toluene as a component of CARC paint, and fine particulate matter relevant to fine sand. Consistent with the multiple and often heavy chemical exposures, a number of studies report that MCS prevalence is greater in GWV than in non-Gulf deployed samples^44,86–90^, making Gulf War veterans a promising group in whom to evaluate the chemical sensitivity relationship to number of SOD2 Ala16 variants. Meanwhile, mitochondrial haplogroup U has been tied to some conditions involving mitochondrial impairment/ oxidative stress^91,92^, and to severity of GWI^93^. In this study, we sought to evaluate whether the SOD2 genetic variant association to chemical sensitivity, reported in the Japanese sample^23^, was supported in a U.S. sample comprising GWV and age-similar nonveterans, focused on the most common ethnicity among Gulf War veterans – white^94^. We especially sought to assess whether the relationship, if present, was more evident, as hypothesized, in the strongly environmentally exposed deployed veteran group and whether a relationship – if any – was influenced by presence of mitochondrial haplogroup U – the second most common haplogroup among white individuals in the U.S.^95^.

##  Results

Participant characteristics are shown in Table 1. Consistent with Gulf-deployed personnel, most participants were male^94^ (>90%). The fraction married in the Gulf deployed sample is materially higher than in the nonveteran sample, chi-squared *p* = 0.030. (This has been noted in other Gulf War studies^34,96^ and was hypothesized to reflect that it is disproportionately veterans with strong social support who may be able to add study participation to their challenging lives^34^). As previously reported for persons of European descent, SOD2 Val16 and Ala16 alleles showed similar prevalence, and the heterozygous state was most common. (Although the heterozygous state appears more common even than the 50% expected, difference from expectation was not significant.) 42% of the veteran group (13/31) cited chemical sensitivity. The 23% in the total sample includes only one additional individual with chemical sensitivity among the nonveteran group (14/60).

**Table 1:** Participant Characteristics

	All (*n* = 60)	Gulf-deployed (*n* = 31)	Nondeployed (*n* = 29)
	*Mean (SD)*	*Mean (SD)*	*Mean (SD)*
Age (years)	56.0 (7.93)	56.1 (7.97)	55.9 (8.04)
# CDC domains	1.42 (1.43)	2.61 (0.92)	0.138 (0.351)
# Kansas domains	2.23 (2.45)	4.26 (1.71)	0.0690 (0.258)
Total Kansas score*	19.1 (22.6)	36.6 (18.7)	0.448 (0.783)

	*N (%)*	*N (%)*	*N (%)*
White	60 (100)	31 (100)	29 (100)
Male	53 (88.3)	28 (90.3)	25 (86.2)
Undergraduate degree	39 (65.0)	17 (54.8)	22 (75.9)
Married	43 (71.7)	26 (83.9)	17 (58.6)
Haplogroup U	13 (21.7)	9 (29.0)	4 (13.8)
SOD2 #Ala16 alleles			
0	12 (20.0)	5 (16.1)	7 (24.1)
1	37 (61.7)	20 (64.5)	17 (58.6)
2	11 (18.3)	6 (19.4)	5 (17.2)
Chemical sensitivity rating > 0	14 (23.3)	13 (41.9)	1 (3.45)
Meet CDC criteria?	28 (46.7)	28 (90.3)	0 (0)
Meet Kansas criteria?	28 (46.7)	28 (90.3)	0 (0)

CDC = Centers for Disease Control and Prevention; SOD2 = Manganese superoxide dismutase, mitochondrial antioxidant; Ala16 = Alanine at amino acid position 16 in the sequence for SOD2.

“N (%)” refers to the number bearing the designated characteristic and the percent of the sample reflected by that N. Presence of qualifying chronic symptoms in at least two of three CDC domains, and at least three of six Kansas domains, are required to meet the respective GWI criteria.

This study focuses on the dominant ethnicity from an ethnically heterogeneous sample (*n* = 92): Of 70 Caucasians, 10 were omitted because of missing relevant data – 7 did not secure genetic data, and 3 did not provide chemical sensitivity ratings. The table presents the 60 participants who provided data for these analyses.

*The Kansas score sums 0–3 ratings (absent, mild, moderate, severe) for the 29 symptoms employed in the Kansas GWI diagnostic criteria, considering symptoms persistent over the prior 6 months (possible range 0–87)^88^.

Table 2 reviews self-rated exposure history of the deployed group, with a strong majority reporting PB use, multiple forms of pesticide exposure, hearing chemical alarms/donning chemical protection gear, inhaling oil fire smoke, and receiving immunizations in theater including receipt of the anthrax vaccine, among other exposures. Although recall accuracy so far after the Gulf conflict may be imperfect and recall bias is possible, it is clear the Gulf-deployed participants were a heavily exposed group.

**Table 2:** Gulf theater exposures for Gulf deployed sample.

Exposure	*N*	# (%)
Heard chemical alarms	31	25 (80.7)
NBC suits	29	24 (82.8)
Gas masks	30	23 (76.7)
Burn pits	28	19 (67.9)
Diesel/petrochemical fumes	29	28 (96.6)
Oil well fires	28	26 (92.9)
Jet fuel burned in tent heaters	25	13 (52.0)
CARC paint	19	8 (42.1)
Struck by depleted uranium fragments	28	0 (0)
Permethrin impregnated uniforms	18	9 (50.0)
Insect repellents	26	17 (65.4)
Sprayed quarters with pesticides	21	6 (28.6)
Saw quarters sprayed with pesticides	22	7 (31.8)
Used pesticides on skin	23	12 (52.2)
Ciprofloxacin	15	10 (66.7)
Pyridostigmine bromide	22	15 (68.2)
Multiple vaccines in arm	27	22 (81.5)
Anthrax vaccine	21	16 (76.2)
Botulinum toxoid vaccine	10	3 (30.0)
Sandstorms	29	25 (86.2)

N = Number providing response for variable; #= Number reporting the specified exposure among of those responding to the query.

Data are based on self-report.

In Table 3, we show the distribution of the number of SOD2 Ala16 alleles as a function of chemical sensitivity level. All of the individuals in the SOD2 VV16 group (i.e. homozygous for valine at position 16) were in the no chemical sensitivity group (the most common group), in the total sample, as well as among deployed individuals. The contingency table with full continuous MCS ratings against number of Ala16 variants is provided in Supplement Table 1 in Supplementary Material 1.

**Table 3:** SOD2 Ala16 allele # as a function of chemical sensitivity level.

Chemical sensitivity level*	Full Sample			Gulf-deployed		
	SOD2 # Ala16 alleles			SOD2 # Ala16 alleles		
	0	1	2	0	1	2
0	12 (26)	27 (59)	7 (15)	5 (28)	11 (61)	2 (11)
1	0 (0)	5 (100)	0 (0)	0 (0)	4 (100)	0 (0)
2	0 (0)	3 (60)	2 (40)	0 (0)	3 (60)	2 (40)
3	0 (0)	2 (50)	2 (50)	0 (0)	2 (50)	2 (50)

SOD2 = Manganese superoxide dismutase, mitochondrial antioxidant; Ala16 = Alanine at amino acid position 16 in the sequence for SOD2. Values in the cells include the number meeting the row and column criteria and the row percent.

*Chemical sensitivity ratings were originally continuous (visual analog scale) but nonzero ratings were split into three levels of successively greater chemical sensitivity, maximizing minimum group number: *N* = 5, 5, 4 in the total sample; *N* = 4, 5, 4 in the deployed sampled. As below, sensitivity analyses were conducted using other choices, such as defining a three-level chemical sensitivity score by splitting nonzero ratings at the median or using a binary chemical sensitivity rating.

GWI case status was unrelated to SOD2 variant status: Logistic regression with robust standard errors, predicting case status by #Ala16 alleles produced a p-value of 0.85 in the total sample and 0.28 in the deployed group separately. This obviates need to adjust for GWI as a confounder.

Table 4 shows the results of ordinal logistic regression analyses (with robust standard errors^97^) for the four-level chemical sensitivity scale. In the group of primary interest, Gulf-deployed veterans (as well as in the total sample), SOD2 Ala16 significantly predicted greater chemical sensitivity. Interest attaches most strongly to the Gulf-deployed group, not solely because of the expected greater power from greater exposure and greater chemical sensitivity prevalence. Also important is that in the deployed group, those without and with chemical sensitivity are drawn from a common base population and common exposure setting in which genetics have scope to operate both to protect against exposure-induced chemical sensitivity and to foster it. The Brant, Score, Wald and Wolfe Gould tests were nonsignificant in this group (*p* = 0.77, *p* = 0.13, *p* = 0.51 and *p* = 0.59, respectively), each indicating that the proportional odds assumption for ordinal logistic regression was not violated. Assessment for significance of an SOD2 (# Ala16 alleles)-by-deployment interaction term (adjusted for both components of the interaction) to evaluate for effect modification by deployment was nominally significant, *p* = 0.039. (Significance of an interaction term supports analysis stratified on the variable interacting with the exposure – here stratified by deployment.) Authority of the test is constrained by the very disparity between the deployed and nonveteran groups which independently underscores appropriateness of separate analysis for the deployed group, because this disparity is also reflected in the fact that not all categories of the outcome are represented in the nondeployed group. In any case, *de facto*, stratified analysis means analysis in the deployed group, since presence of just one chemically sensitive individual in the nonveteran group precludes separate analysis in that group.

**Table 4:** SOD2 Ala16 allele # predicts chemical sensitivity.

Group	*N*	Ordinal logit, unadjusted			Ordinal logit, adjusted (haplogroup U)		
		OR (SE)	95% CI	*P*	OR (SE)	95% CI	*P*
All	60	3.56 (1.83)	1.30, 9.74	0.013	4.07 (1.99)	1.56, 10.6	0.004
Gulf-deployed	31	6.36 (4.85)	1.42, 28.4	0.015	6.68 (5.06)	1.51, 29.5	0.012

SOD2 = Manganese superoxide dismutase, mitochondrial antioxidant; Ala16 = Alanine at amino acid position 16 in the sequence for SOD2; N = number; OR = odds ratio; SE = standard error; CI = confidence interval; P = probability.

Ordinal logistical regression (robust standard errors) assessing the four-level chemical sensitivity rating as the outcome, with SOD2 (# Ala16 alleles) as predictor.

Repeating the regression analysis with adjustment for GWI case status nominally strengthened the p-values for the relationship of SOD2 # Ala16 alleles to chemical sensitivity (*p* = 0.004 and *p* = 0.005 for the full sample and the deployed sample, respectively), but tests for proportional odds could not be conducted, so the assumption could not be formally assessed or confirmed. Of note, GWI case status was a separately significant predictor.

Adjustment for mitochondrial haplogroup U did not materially alter significance (which was minimally strengthened) in regression analyses, and haplogroup U was not an independently significant predictor in the total sample or in Gulf-deployed veterans separately.

Sensitivity analyses included a three-level model (splitting nonzero chemical sensitivity ratings at the median), which also showed significance for the SOD2 Ala16 allele number as a predictor of chemical sensitivity (Supplement Table 2 in Supplementary Material 1). Dichotomizing chemical sensitivity as present or absent also yielded statistical significance of SOD2 Ala16 allele number as a predictor of chemical sensitivity (logistic regression, robust standard errors).

Nonparametric tests of trend, including Kendall’s Tau and Spearman’s Rank Correlation Coefficient, also affirm significance of SOD2 Ala16 as a predictor of chemical sensitivity severity for the four-level and three-level chemical sensitivity outcomes (Table 5). Significance of the SOD2 Ala16 relation to chemical sensitivity in these nonparametric assessments helps ensure that model fit or stability issues are not the foundation for the significance in the ordinal logit models.

**Table 5:** Nonparametric sensitivity analyses: SOD2 Ala16 allele # relates to chemical sensitivity severity.

Group	*N*	Kendall’s Tau		Spearman’s Rank	
		Tau-a	*P*	Spearman’s rho	*P*
Four Category					
All	60	0.13	0.024	0.29	0.024
Gulf-deployed	31	0.23	0.0147	0.44	0.014
Three Category					
All	60	0.13	0.0199	0.30	0.021
Gulf-deployed	31	0.25	0.0096	0.46	0.009

N = Number; P = Probability. Additional significant digits are retained in those p-values where rounding could propagate to a difference of the second post-decimal value.

## Discussion

In this sample of persons of European descent, among those who mostly had heavy chemical exposure (as a result of 1990–1991 Gulf theater deployment), the SOD2 Ala16 allele significantly predicted greater likelihood/severity of chemical sensitivity. Among those without similar exposure history, the nonveteran subgroup in whom chemical sensitivity was rare (one person affected), a similar relationship could not be shown. This coheres with the observation that management of mitochondrial oxidative stress will be selectively important where there is (more) mitochondrial oxidative stress to manage, as would be expected in settings like chemically induced oxidative stress and mitochondrial impairment^34^ (with the mitochondrial impairment itself promoting further oxidative stress^3–5^).

Findings have a suggested precursor in the prior study of Japanese paper pulp company workers in which presence of the SOD2 Ala16 allele was also significantly related to chemical sensitivity, and – as here – particularly to more severe chemical sensitivity^23^. In East Asia, the Val16 allele has 86.2% reported prevalence^12^, suggesting different selection pressures in that population. Our (priority) deployed sample and the Japanese paper pulp worker sample each focus on participants without and with chemical sensitivity from a common exposure setting and include examination of severity levels of chemical sensitivity as a function of genetics, both finding that the Ala16 or AA16 predominance is principally evident in those with relatively severe chemical sensitivity. These two studies provide provisional support for regulation of mitochondrial oxidative stress (or potentially more specifically, mitochondrial conversion of superoxide to hydrogen peroxide) in the pathogenesis of chemical sensitivity. This need not exclude involvement of other pathogenic mechanisms. Additionally, since the SOD2 impact is expected to be relevant preferentially where there is significant mitochondrial oxidative stress (e.g. in a significant exposure context – i.e. a gene-environment interaction), we do not conclude that SOD2 genetics are irrelevant to chemical sensitivity in nonveterans, just that the ability (power) to see a relationship in a low exposure/low MCS prevalence group will be compromised.

Mitochondrial impairment engenders increased mitochondrial oxidative stress (and vice versa)^3–5^. No separate effect of haplogroup U was observed in our sample, but the issue of whether there might be an interaction between SOD2 genetics and mitochondrial haplogroup might merit reassessment in a larger sample.

An Italian study also assessed SOD2 Val16 vs. Ala16 alleles and, though claiming to accord with the Cui study (Japanese paper pulp company), in fact reported a predominance of Val16 alleles in association with chemical sensitivity, that approached statistical significance, just over *p* = 0.050^13^. (This p-value was from our chi-squared analysis of their presented data, as no corresponding p-value was provided.) Unlike our deployed sample and the Japanese study above^23^, this study was limited by the fact that their MCS and no-MCS groups did not come from a common similarly-exposed sample. Indeed, the MCS group was drawn from a hospital patient sample, which could introduce Berkson’s bias, as being a hospital patient may be subject to different genetic selection influences. (MCS itself is unlikely to have led to hospitalization, though what is meant by hospital patient sample is not clear.) Moreover, the no-MCS group was not selected for having had chemical exposure: Only with exposure can genetic factors be expected to exert an influence on *absence* of chemical sensitivity induction, so choice of the no-MCS group may also influence genetic relationships identified. Additionally, that study did not examine SOD2 AV16 association to MCS severity. Intriguingly, however, potentially in line with our and the Japanese samples’ finding, they did report that oxidative stress was greatest in the MCS subsample that was homozygous for SOD2 Ala16. This was the genetic group most tied to severe MCS in our deployed sample and the Japanese sample^98^.

Mitochondrial oxidative stress management holds particular promise for explaining the peculiar features of MCS, and in particular, the typical presence of a triggering exposure event following which previously tolerated low-level chemical exposures are problematic. Mitochondrial DNA is vulnerable to oxidative injury and has relatively deficient repair mechanisms^99^, and mitochondrial impairment – indeed whether from oxidative DNA, RNA, or potentially protein or lipid injury – can lead to ongoing impaired energy production and increased oxidative stress. Mitochondrial oxidative stress that is inadequately managed by mitochondrial antioxidant defenses can therefore set individuals up with ongoing energy compromise and heightened free radical production. This, in turn, can perch them closer to, or even on, the steep part of the curve tying cell energy impairment and/or oxidative stress to symptoms. Thus, even a low level added exposure can tip them into new or worsened symptoms: This is because many or most chemicals – regardless of chemical class – confer toxicity in part through mitochondrial and oxidative mechanisms^7^. Although this positions mitochondrial oxidative stress management in a pivotal role for the triggering event, resulting ongoing elevated free radical production can mean that subsequent oxidative stress – not necessarily mitochondrial oxidative stress – may suffice to surpass antioxidant defenses and induce symptoms. This explanation also accounts for the fact that among Gulf War veterans, it is disproportionately those who developed chronic multisymptom illness (GWI), for which severity is tied to severity of mitochondrial compromise^34^, in whom new-onset chemical sensitivity arises.

A hypothesis in which mitochondrial oxidative stress (and its management) is important in MCS induction could in principle accommodate either an Ala16 or Val16 predominance with third factors determining which is more problematic. SOD2 converts mitochondrial superoxide to hydrogen peroxide, which itself is not benign and can be converted to the highly damaging hydroxyl radical, especially in the presence of iron (Fenton reaction), if not first converted to water by catalase, glutathione peroxidase or peroxiredoxins^100^. It has been observed that “increases in SOD2 expression promote oxidative stress”^22^. Particularly where iron is abundant, SOD2 can incorporate iron in lieu of manganese converting from an antioxidant form to a prooxidant peroxidase^22^. FeSOD2 is reported to cause mitochondrial dysfunction in vivo (as well in vitro)^22^. This could provide one potential basis for the apparent difference between our finding and the Cannata finding since persons of British/Irish/Scottish descent have high carrier status for hemochromatosis (called the “Celtic Curse”^101^) that can increase iron levels^98^, and the US-Caucasian population – unlike the Italian one – is heavily descended from this group. This could provide one potential basis for the apparent different genetic predisposition in the two populations. Also notable is that increased production of hydrogen peroxide has potential for a positive feedback impact, since peroxiredoxins, which help to clear hydrogen peroxide, are themselves inactivated in the setting of high hydrogen peroxide levels^102,103^. Indeed, it is possible that the signaling functions of hydrogen peroxide (and redox state) could be involved in MCS pathogenesis – in lieu of or in addition to involvement of oxidative injury per se: Oxidative stress has many critical functions, including signaling for apoptosis that can help to clear cells that may have ceased to contribute to function while continuing to exert nutrient and energetic demands; and potentially to enhancing cell barrier via oxidation of membrane lipids. Other factors beyond those above could influence which genetic variant is more adverse. For instance, where there is more nitric oxide to bind superoxide and result in damaging peroxynitrite, the Val16 allele may be more problematic (less superoxide conversion to hydrogen peroxide). It would be desirable to secure a better understanding of the factors and settings that lead to differential adverse consequences for Val16 vs. Ala16 alleles, in the setting of MCS as well as outside of it. We speculate that the factors noted here, and perhaps particularly iron status, may prove important.

Others have identified mechanisms relevant to chemical sensitivity that may derive from and/or have their toxicity amplified by mitochondrial oxidative stress and associated mitochondrial impairment, such as elevated peroxynitrite (which is formed from superoxide plus nitric oxide – **NO**)^104,105^ and elevated *N*-methyl-D-aspartate (**NMDA**) activity^106,107^. (Neuronal NO synthase (**nNOS**) is associated with NMDA receptors in the brain^108^, and calcium flux through NMDA receptors stimulates nNOS to produce NO^109^, which can diffuse into the mitochondrion and bind with superoxide to form peroxynitrite – or can bind to other sources of superoxide outside the mitochondrion to do so.) Exposures that have been reported to trigger onset of chemical sensitivity, such as organophosphate and carbamate pesticides^110,111^, cohere with these findings in that organophosphates and carbamates mediate their toxicity considerably through mitochondrial and oxidative stress mechanisms^46,48,51,112,113^. Other reported triggers of chemical sensitivity include metals (e.g. as implants)^114^, which also mediate toxicity through oxidative and mitochondrial mechanisms^115–132^. Mitochondrial injury and resulting increased oxidative stress may be expected to magnify the impact of subsequent exposures that confer potential toxicity through oxidative and mitochondrial mechanisms, enhancing magnitude of mitochondrial defects and competition for antioxidant defenses. Increased shortfall in mitochondrial energy production and/or demand for antioxidant defenses, arising from competition for such defenses, may be most difficult to redress in those bearing genetic features that adversely alter the balance of mitochondrial oxidative stress to antioxidant defense.

The observed elevation in chemical sensitivity associated with altered mitochondrial oxidative stress management aligns with recent evidence implicating compromised mitochondrial respiratory chain function in GWI^34^. In settings where this is coupled with altered management of mitochondrial antioxidation, chemical influences on mitochondrial energy production and oxidative stress production may be particularly problematic. It is in just such settings that adverse mitochondrial oxidative stress management may be expected to lead mitochondrial antioxidant defenses to be overwhelmed sufficiently to precipitate clinical symptoms. According to this rationale, patients with chemical sensitivity beyond veterans with Gulf War illness might also be predicted to have increased prevalence of mitochondrial impairments, magnifying the impact of mitochondrial oxidative stress.

Our sample was small relative to many samples used for genetic assessment – but power is not a function of sample size alone. Inclusion of GWV with a strong history of significant (mitochondrially toxic) chemical exposure, with high rates of chemical sensitivity related to such exposure^87^, and with known mitochondrial compromise (among those with GWI)^33,34^, augments expected power to identify genetic relations of mitochondrial oxidative stress management to chemical sensitivity.

To clarify, the relation of a gene involved in regulation of mitochondrial oxidative stress to chemical sensitivity can be best assessed in samples that have considerable mitochondrial oxidative stress to regulate, due to significant chemical exposure – and that have sufficiently high rates of resulting chemical sensitivity to allow examination of gene variant relations to that outcome. Replication is desirable, and indeed this paper represents a replication – agreeing with the only other study in which assessment occurred with both MCS and no-MCS individuals drawn from a common/shared chemically-exposed sample, in which there are ~ high attendant rates of MCS. More important than sheer sample size for future studies will be ensuring that these critical conditions are met, as these are likely critical for adequate study power. Assuming these prerequisites are met, conducting a replication would be valuable, and a larger sample size is desirable to offer additional statistical power and authority.

In a sense, our sample constitutes the reassessment of the relationship in an independent chemically exposed sample with high chemical sensitivity rates – affirming the Cui findings, but further independent and adequately powered samples (not considering sample size alone) are always welcome. Restriction to one ethnicity is a strength and a weakness: Findings need not relate to other ethnicities that may differ in allele frequencies, and in other genes and exposures that may moderate the relation of SOD2 alleles to outcomes. Future work should assess whether these gene-exposure relationships extend to broader or different cohorts. Findings from this study cohere with the burgeoning body of evidence implicating mitochondrial and oxidative mechanisms in toxicity of numerous exposures spanning many different exposure classes and nominal mechanisms of action^7,133^, which adds triangulating support for a link between mitochondrial oxidative stress management and chemical intolerance. We include an ordinal logistic regression model for which rules of thumb recommend a minimum of five observations per outcome level while our minimum observation number per level is four: However, sensitivity analyses including nonparametric Kendall’s Tau and Spearman’s Rank Correlation Coefficients support the observed significance and help assure that factors like model instability were not the foundation for significance of observed relationships. (Inclusion of ordinal logistic regression allowed for assessment adjusting for haplogroup U: The SOD2 chemical sensitivity relation was preserved with haplogroup U (which was not itself significant) – however, nonparametric analysis shows that the fundamental finding tying SOD2 Ala16 variants to greater risk/severity of chemical sensitivity does not rest on the ordinal logistic regression model alone.)

There has been dispute regarding whether MCS is physiologically based versus a somatoform disorder, with the latter reflected in the ICD-10 code F45 used for multiple chemical sensitivity^2^. (Somatoform disorders are conditions believed to be psychological in origin that manifest as physical symptoms but for which a recognized physiological basis is not understood^134^. Of note, somatoform disorders are officially termed somatic symptom disorders.) The SOD2 genetic findings appear to position chemical sensitivity in the realm of a physiologically-mediated condition, contravening presumptions of a psychological foundation. Moreover, these findings implicate a plausible mechanism by which many chemicals of diverse chemical classes might, in vulnerable individuals, lead to symptoms^7,133^. Because mitochondrial impairment does not fit an organ-based pattern, and testing for mitochondrial impairment is not widely available, conditions tied to such impairment are especially subject to potentially erroneous presumptions that psychogenic foundations are operating.

## Methods

### Ethics statement

Data acquisition was funded by the Department of Defense Congressionally Directed Medical Research Programs (**GW130106**). The study funders had no role in the study design, collection, analysis and interpretation of data, or the decision to publish. The study was approved by the UCSD Human Research Protections Program (protocol #151293) and the DoD Human Research Protections Office (log # A-19535.a), and all participants gave written informed consent. All methods were performed in accordance with the relevant guidelines and regulations.

### Participants

Sixty white participants, including 31 veterans deployed to the 1990-91 Gulf theater and 29 healthy nonveterans, completed nuclear as well as mitochondrial DNA assessments and rated chemical sensitivity.

Participants were scored by Centers for Disease Control and Prevention (**CDC**)^135^ and Kansas^88^ Gulf War illness symptom criteria, with 28 veterans qualifying as having Gulf War illness by both criteria (and no nonveterans meeting Gulf War illness symptom criteria). Both sets of criteria require deployment to the Gulf any time between August 1, 1990, and July 31, 1991, inclusive. Both require persistent symptoms, for at least six months, arising during or after Gulf deployment. Kansas criteria require that these symptoms were multiple and/or at least moderate in severity in at least 3 of the 6 Kansas domains of fatigue/sleep, pain, neurological, gastrointestinal, respiratory and dermatologic^88^. CDC criteria require that at least one such symptom, of any severity, be present in at least two of the three domains of fatigue, mood/cognitive, and musculoskeletal^135^. For nonveterans, symptoms were scored irrespective of Gulf deployment or timing of symptom onset.

Exclusions that applied to all individuals encompassed medical conditions (such as lupus, multiple sclerosis) that could produce symptoms that could be confused for GWI, whether or not such symptoms were present. Additionally, persons were excluded if retrospective self-rating of health prior to Gulf participation (veterans) or 1990 (nonveterans) was less than “very good” or “excellent” – to exclude among Gulf War veterans those who may have had health problems prior to Gulf deployment that might be responsible for current health problems (if present) and to provide for comparability of nonveterans on general health to deployed veterans at that time. Nonveterans were selected to meet *neither* Kansas nor CDC GWI symptom criteria to aid comparability to nonveterans included in prior studies of Gulf War illness.

Participants were recruited from past Gulf War studies that included both Gulf War veterans and healthy nonveterans, with healthy nonveterans typically selected to have matched to a prior Gulf War veteran participant on age, sex and ethnicity. Our rosters of past study participants were complemented by additional recruitment using social media and, particularly for nonveterans, ResearchMatch. Both Gulf War veterans and nonveteran participants were recruited primarily from the Southern California area.

The sample is focused on Americans of European descent (i.e. white), based on the following considerations: Ability to detect genetic relations to outcomes is advantaged by restriction of ethnic heterogeneity^136^, so we focus on the majority ethnicity among Gulf War veterans^137^. Underscoring this are not merely ethnic disparities in minor allele frequencies for SOD2 AV16^12^, but in the dominant allele, that may mean mixing ethnicities could obviate ability to identify relationships. Finally, the interest in assessing mitochondrial haplogroup U served as a further impetus, as, in the United States, this haplogroup is mostly seen in non-Hispanic whites^95^.

### Rationale for inclusion of nonveterans

Inclusion of nonveterans provides for a group without deployment-associated or other military exposures, which are frequently present in veterans even among those who served stateside or in locations like Europe. (Such exposures can include e.g. pesticides, vaccines, jet fuels, cleaning and restocking of used nuclear/biological/chemical protection suits, burn pits, etc.) Those who are Gulf-era but not Gulf-deployed were less healthy as a group, as unhealthy persons are not selected for deployment to high threat areas^138^; i.e. many had reasons for nondeployment. Moreover, as above, many military personnel who were not Gulf-deployed had multiple military exposures, even if not the full set experienced in the Gulf theater. Inclusion of a nonexposed group may be important for ascertaining whether a genetic relationship of SOD2 AV16 to MCS (if present) is expressed differentially in those from a high exposure environment. Thus, choice of age-similar healthy nonveterans aids the intent to evaluate whether genetic predictors relevant to detoxification (with SOD2 considered a phase 2 detoxification gene^139^) or more specifically to management of mitochondrial oxidative stress, predestines to illness (as genetic factors do for some heritable conditions) or whether the impact is relatively conditional on an exposure setting in which a need for the function of the gene product is magnified – as is presumed for chemical sensitivity.

### Genetic assessments

Genetic assessments were performed by commercial laboratories, as follows.

#### Nuclear DNA assessment

SOD2 Ala16 vs. Val16 allele status and allele calling was performed by 23andMe (Version 5). 23andMe version 5 employs the Illumina Infinium Global Screening Array, supplemented with ~ 50,000 variants of custom content^140^. The Illumina Global Screening Array combines curated clinical research variants for precision medicine research^141^. Saliva was sent to 23andMe, where all testing was performed in a CLIA certified laboratory. Samples underwent heat inactivation to reduce enzymes that could contaminate the samples. DNA was extracted and amplified, and DNA was applied to the chips which contain probes that hybridize the DNA. The chips were placed in hybridization ovens allowing DNA to adhere to chips. Chips were stained and washed to remove impurities, then fed into machines for the final readout^142^. Data were provided in VCF files coded as rs numbers. The rs number is a unique identifier used to code single nucleotide polymorphisms (SNPs) in genetic data, and rs4880 references the t vs. c. (i.e. A vs. G) substitution at nucleotide 47 in the genetic sequence for SOD2, leading to the valine vs. alanine amino acid substitution at position 16 in the amino acid sequence. SOD2 allele status was shared by 23andMe as 1,0,0 (corresponding to homozygous A referencing valine); 0,1,0 (referencing heterozygous status); or 0,0,1 (corresponding to homozygous G referencing alanine). This was coded by us for use in analysis as the number of Ala16 alleles – 0, 1 or 2.

#### Mitochondrial haplogroup assessment

Mitochondrial haplogroup was determined by GeneDx, from full mitochondrial genome sequencing conducted on provided phlebotomy samples (two 4 mL purple top EDTA tubes for a total of 8 mL of whole blood). GeneDx did the haplogroup calling. GeneDx conducts sequence analysis (and deletion testing) of the entire mitochondrial genome, analyzed using high throughput “next generation” sequencing technology to achieve efficiency with high sensitivity. “The mixture of two different Whole Mitochondrial Genome Amplification Amplicons from genomic DNA by long-range PCR are sequenced using a novel solid-state sequencing-by-synthesis process that allows sequencing a large number of DNA fragments in parallel. With respect to using two different Whole Mitochondrial Genome Amplification Amplicons, two different sets of non-overlapping primers from the D-loop region are used separately, such that each can amplify the entire mitochondrial genome to ensure every single base of the mitochondrial genome can be sequenced correctly without being affected by the primer sequence. Because the D-loop region of the mitochondrial genome is never deleted, all of the mitochondrial DNA molecules with deletions are also amplified. DNA sequences are assembled and compared to the published mitochondrial genome reference sequences for analysis…A reference library of more than 6000 samples from different ethnic groups and online databases for mtDNA variations [is employed]”^143^. GeneDx further shared that “The average coverage (number of reads) for every nucleotide of the mitochondrial genome of a specific sample ranged from 5,000× to 25,000× for different samples, with a standard deviation of ≤ 10% of the average coverage. The coverage for nucleotide positions within 200 bp from the primer regions at the beginning and end of the mitochondrial genome could be lower due to removal of the primer sequences during alignment”^144^.

Standard approaches to haplogroup assessment involving markers within the mitochondrial genome are widely known^145^. Mitochondrial DNA haplogroup analysis was performed by GeneDx for each participant with all homoplasmic mitochondrial DNA variants using an online tool^146^. This site references phylotree.org to define the variants that determine haplogroup. GeneDx provided the mitochondrial haplogroup letter designation, which was recoded by us as haplogroup U absent (0) or present (1).

### Chemical sensitivity

Chemical sensitivity was self-rated using a single-item 0–10 visual analog scale. This has been validated against the chemical sensitivity query from the Kansas Gulf War illness diagnostic instrument^88^: A previously published correlation of *r* = 0.57, *p* = 0.0001^7,147^ was based on cases only, from that sample. Employing the combined case-control sample, the value is *r* = 0.71, *p* < 0.0001. The timeframe, scoring and question used for the Kansas instrument differ (six months timeframe; 4 point Likert scale of absent, mild, moderate or severe; inquiring about “having physical or mental symptoms after breathing in certain smells or chemicals”). The UCSD query is more specific in asking the participant to characterize themselves as having “unusual sensitivity,” relative to others, asking people to rate 0–10, prior two weeks “Chemical sensitivity (e.g., unusual sensitivity to smells).” Moreover, the UCSD chemical sensitivity measure performs better than the Kansas chemical sensitivity score when gauged against a measure of actual drug and chemical adverse effect propensity that assesses the fraction of actually experienced exposures (across a large number evaluated) to which adverse effects were experienced: *r* = 0.41, *p* = 0.0081 for the UCSD chemical sensitivity score vs. adverse effect propensity; *r* = 0.31, *p* = 0.047 for the Kansas chemical sensitivity score vs. adverse effect propensity^7^. For purposes of analysis, to foster adherence to ordinal logistic regression assumptions, chemical sensitivity ratings were collapsed into four ordered categories (levels) with similar numbers of participants in the nonzero categories to maximize the minimum number per category.

For sensitivity analysis purposes, a three-value chemical sensitivity rating split nonzero ratings at the median value. Finally, a binary chemical sensitivity rating considered only presence or absence of chemical sensitivity (i.e. original rating 0 or > 0).

Since all 60 included participants had nuclear and mitochondrial DNA assessment and provided a chemical sensitivity rating, there were no missing data to be addressed.

### Analysis

Descriptive statistics were used to portray participant characteristics, as well as distribution of SOD2 Ala16 allele number, haplogroup U, and chemical sensitivity in the full sample and in Gulf-deployed veterans separately. GWI case status was assessed for a relation to SOD2 Ala16 allele number to determine if it qualified as a potential confounder. (Absence of an observed relationship in the total or deployed sample would obviate need to adjust for GWI.) Ordinal logistic regression with robust standard errors evaluated prediction of chemical sensitivity by SOD2 Ala16 allele number in the total sample and in the sample of principal interest, deployed veterans. The Brant test (also the Score, Wald and Wolfe Gould tests) assessed for violation of proportional odds assumptions for this sample.

Sensitivity analyses reassessed the relationship, using the three-level chemical sensitivity variable (ordinal logistic regression with robust standard errors) and the binary chemical sensitivity variable (logistic regression with robust standard errors) as the outcome variable. Each analysis was performed in the total sample and in Gulf War veterans separately. Each was conducted without and with adjustment for mitochondrial haplogroup U. Effect modification by deployment (i.e. by chemical exposure setting) was evaluated via assessing significance of an interaction term in the regression analysis, defining the interaction as deployment-by-SOD2 Ala16 allele number, adjusted for the individual terms in the interaction. Significance of the interaction term signifies effect modification and adds support to separate analysis of the deployed sample.

As additional validation of results, analyses for the four- and three-level chemical sensitivity relationships were reassessed using nonparametric ordered trend tests (Kendall’s Tau and Spearman’s Rank Correlation Coefficients) to ensure against factors such as model instability as a possible source of significance (if observed) in the ordinal logit model.

Analyses were conducted using Stata/SE versions 8.0 and 18.0. P-values < 0.05 designated statistical significance.

## Electronic supplementary material

Below is the link to the electronic supplementary material.

Supplementary Material 1

Supplementary Material 2

## Data Availability

Genetic data are available at dbSNP (# ss2137544501, https://www.ncbi.nlm.nih.gov/projects/SNP/snp_viewBatch.cgi?sbid=1063569). Genetic data are also available in Supplementary Material 2, together with the chemical sensitivity outcome. Statistical output and other data are available on request to the corresponding author (bgolomb@ucsd.edu).
