# Supplementary material for: SOD2 genetics regulating mitochondrial management of oxidative stress is tied to chemical sensitivity in Gulf war veterans
Source: Sci Rep. 2025 Jul 8;15:24418. doi: 10.1038/s41598-025-09916-w (PMC12238362; doi:10.1038/s41598-025-09916-w)
Supplement: Supplementary file 1 — Supplementary Material 1 [file 41598_2025_9916_MOESM1_ESM.docx]

**Supplemental Material**

| **Supplement Table 1**. SOD2 Ala16 allele # as a function of 0-10 chemical sensitivity rating | | | | | | | |
| --- | --- | --- | --- | --- | --- | --- | --- |
| **Full Sample** | | | | **Gulf-deployed** | | | |
| **Chemical sensitivity** | **SOD2 Ala16 allele #** | | | **Chemical sensitivity** | **SOD2 Ala16 allele #** | | |
|  | **0** | **1** | **2** |  | **0** | **1** | **2** |
| 0 | 12  (26) | 27  (59) | 7  (15) | 0 | 5  (28) | 11  (61) | 2  (11) |
| 1 | 0  (0) | 1  (100) | 0  (0) | 1 | 0  (0) | 1  (100) | 0  (0) |
| 2 | 0  (0) | 1  (100) | 0  (0) | 2 | 0  (0) | 1  (100) | 0  (0) |
| 3.6 | 0 (0) | 1  (100) | 0 (0) | 3.6 | 0 (0) | 1  (100) | 0 (0) |
| 4.9 | 0 (0) | 1 (100) | 0 (0) | 4.9 | 0 (0) | 1 (100) | 0 (0) |
| 4.9 | 0  (0) | 1  (100) | 0  (0) |  | | | |
| 5 | 0 (0) | 1  (100) | 0 (0) | 5 | 0 (0) | 1  (100) | 0 (0) |
| 5.8 | 0  (0) | 1  (100) | 0 (0) | 5.8 | 0  (0) | 1  (100) | 0 (0) |
| 6 | 0  (0) | 1  (100) | 0 (0) | 6 | 0  (0) | 1  (100) | 0 (0) |
| 6.4 | 0  (0) | 0  (0) | 1  (100) | 6.4 | 0  (0) | 0  (0) | 1  (100) |
| 6.6 | 0  (0) | 0  (0) | 1 (100) | 6.6 | 0  (0) | 0  (0) | 1 (100) |
| 7.1 | 0  (0) | 1  (100) | 0  (0) | 7.1 | 0  (0) | 1  (100) | 0  (0) |
| 9 | 0 (0) | 0  (0) | 1 (100) | 9 | 0 (0) | 0  (0) | 1 (100) |
| 9.9 | 0  (0) | 1  (100) | 0 (0) | 9.9 | 0  (0) | 1  (100) | 0 (0) |
| 10 | 0  (0) | 0  (0) | 1  (100) | 10 | 0  (0) | 0  (0) | 1  (100) |

| **Supplement Table 2a**. SOD2 Ala16 allele # predicts three-category and two-category chemical sensitivity rating. | | | | | | | | | |
| --- | --- | --- | --- | --- | --- | --- | --- | --- | --- |
| **Group** | **N** | **Three category chemical sensitivity outcome*** | | | | | | | |
|  |  | **Ordinal logit, unadjusted** | | | **Ordinal logit, adjusted**  **(haplogroup U)** | | | **Chi-squared** | |
|  |  | **OR (SE)** | **95% CI** | **P** | **OR (SE)** | **95% CI** | **P** | **Z** | **P** |
| All | 60 | 3.83 (2.14) | 1.29, 11.4 | 0.016 | 4.76 (2.67) | 1.59, 14.3 | 0.005 | 13.1 | 0.011 |
| Gulf-deployed | 31 | 9.07 (9.21) | 1.24, 66.3 | 0.030 | 10.6 (10.7) | 1.48, 76.3 | 0.019 | 12.3 | 0.015 |
|  |  | **Two category chemical sensitivity outcome**† | | | | | |  | |
| **Group** | **N** | **Logit, unadjusted** | | | **Logit, adjusted**  **(haplogroup U)** | | |  |  |
|  |  | **OR (SE)** | **95% CI** | **P** | **OR (SE** | **95% CI** | **P** |  |  |
| All | 60 | 3.03 (1.34) | 1.27, 7.21 | 0.013 | 3.70 (1.74) | 1.47, 9.28 | 0.005 |  |  |
| Gulf-deployed | 31 | 4.97 (3.69) | 1.16, 21.3 | 0.031 | 5.96 (4.60) | 1.32, 27.0 | 0.021 |  |  |
| *Ordinal logit uses robust standard errors. Three category chemical sensitivity outcome splits non-zero chemical sensitivity ratings at the median. The three categories encompass original 0-10 ratings of: 0; 1 to <6; 6-10 – now assigned values of 0, 1, and 2 respectively.  †Logistic regression (robust standard errors) translates original 0-10 chemical sensitivity ratings to values of 0 (if the original rating was zero) and 1 (if the original rating was >0 – *de facto* 1-10 inclusive). | | | | | | | | | |

| **Supplement Table 2b**. SOD2 Ala16 allele # as a function of the three-category chemical sensitivity rating. | | | | | | | |
| --- | --- | --- | --- | --- | --- | --- | --- |
| **Full Sample** | | | | **Gulf-deployed** | | | |
| **Chemical sensitivity** | **SOD2 Ala16 allele #** | | | **Chemical sensitivity** | **SOD2 Ala16 allele #** | | |
|  | **0** | **1** | **2** |  | **0** | **1** | **2** |
| 0 | 12  (26) | 27  (59) | 7  (15) | 0 | 5  (28) | 11  (61) | 2  (11) |
| 1 | 0  (0) | 7  (100) | 0  (0) | 1 | 0  (0) | 6  (100) | 0  (0) |
| 2 | 0  (0) | 3  (43) | 4  (57) | 2 | 0  (0) | 3  (43) | 4  (57) |
